# Unusual Etiology and Diagnosis of Oroantral Communication due to Late Implant Failure

**DOI:** 10.1155/2017/2595036

**Published:** 2017-10-03

**Authors:** Rabah Nedir, Nathalie Nurdin, Marion Paris, Marc El Hage, Semaan Abi Najm, Mark Bischof

**Affiliations:** ^1^Ardentis Clinique Dentaire Vevey, Swiss Dental Clinics Group, Rue du Collège 3, 1800 Vevey, Switzerland; ^2^Ardentis Clinique Dentaire Morges, Swiss Dental Clinics Group, Rue Saint-Louis 2B, 1110 Morges, Switzerland; ^3^Ardentis Clinique Dentaire Lausanne, Swiss Dental Clinics Group, Voie du Chariot 6, 1003 Lausanne, Switzerland; ^4^Ardentis Clinique Dentaire Geneva, Swiss Dental Clinics Group, Rue Thomas-Masaryk 1, 1202 Geneva, Switzerland

## Abstract

Oroantral communication (OAC) rarely occurs long after implant placement. The present report describes the rare etiology and the difficulty of the diagnosis of an uncommon OAC occurring 10 years after the implant placement in the posterior maxilla. The difficulty of the diagnosis lies in the absence of clinical symptoms of sinusitis and presence of multiunit prosthesis hiding implant failure. This case report supports the need for sinus check-up during a routine implant examination.

## 1. Introduction

Oroantral communication (OAC) is a pathological connection between the oral cavity and the maxillary sinus due to loss of soft and hard tissues that normally separated these compartments. The OAC is often confused with the oroantral fistula (OAF) which is defined as a persistent epithelialized open communication [1]. OAC and OAF occur most frequently as a result of maxillary posterior tooth extraction (92.63%), followed by pathological lesions in the sinus (presence of cysts and tumors; 4.47%) and trauma (1.30%). Periodontal infections are the cause in only 0.93% of cases, with other factors accounting for 0.65% [2–4]. OAC complications may occur early after implant placement but rarely long after, and it rarely concerns osseointegrated implants [5].

Patients with OAF are generally prone to sinus infections. Complications include sinusitis and, in rare cases, pansinusitis, cerebral thrombophlebitis, and brain abscess. About 50% of sinusitis occurs on the third day after the manifestation of the OAC [6]. This infection is most often acute and needs to be treated with emergency cares. The clinical diagnosis of sinusitis is generally characterized by the following symptoms [7]: facial pain, facial pressure, facial congestion, nasal congestion, nasal obstruction, nasal discharge, purulence or discolored postnasal drainage, hyposmia or anosmia, fever, purulence on intranasal examination, headache, halitosis, fatigue, dental pain, cough, ear pain, and ear pressure. For the treatment, it is necessary to completely eliminate any type of sinus infection before the closure [8]. After acute biomaterial-related sinusitis, when the implant was placed with sinus elevation and grafting, care involves antibiotic therapy, sinus endoscopy, surgical exploration, removal of all infected bone graft, potential removal of the implant, restoration of proper drainage, and ventilation of the sinus [9–12].

This report describes the difficulty in diagnosing the late failure of one implant under a stable bridge placed 10 years after implant placement. The chronic sinusitis related to uncommon OAC was asymptomatic; the patient did not complain and did not show any intraoral and extraoral clinical symptoms.

## 2. Case Presentation

**Figure 1:**
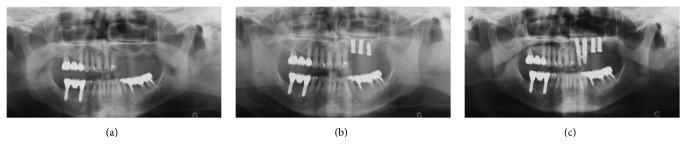
Placement of implants, panoramic radiographs. (a) Initial situation, (b) immediately after implant placement in sites 23, 24, and 25, and (c) 7 months later, immediately after implant placement in sites 25 and 26.

In April 2003, a 62-year-old Caucasian woman presented for the rehabilitation of sites 23–26 (residual bone height: 12, 5, 2, and 4 mm, resp.; Figure 1(a)). She required implant placement to support a fixed partial denture. Her general medical history did not reveal any particular problem and her dental history showed that she had been treated for periodontal disease. She did not suffer from chronic maxillary sinus disease. A lateral sinus floor augmentation with deproteinized bovine bone material (Bio-Oss®, Geistlich AG, Wolhusen, Switzerland) was performed with the simultaneous placement of three standard endosseous implants (≤10 mm in length; Straumann AG, Basel, Switzerland) in sites 23–25 (Figure 1(b)). Two months later, implant 25 was removed because of mobility. In November of the same year, two implants (10 mm in length) were placed in sites 25 and 26 by using lateral window and osteotome technique with a membrane (Bio-Gide®, Geistlich Pharma AG) and grafting material (Bio-Oss, Geistlich Pharma AG; Figure 1(c)). After 4 months, a percussion test showed that all implants were clinically stable. They were resistant to tightening with a 35-N·cm torque; they were functionally loaded with a screw-retained fixed partial denture (FPD). No postoperative acute sinusitis or another complication was reported by the patient.

**Figure 2:**
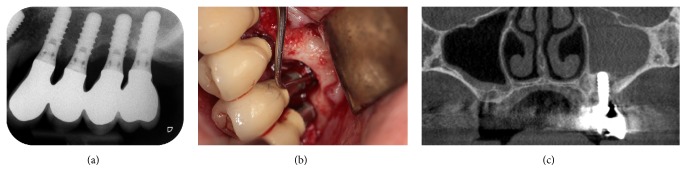
Ten years after implant placement. (a) Periapical radiograph, (b) clinical view, flap at site 25, and (c) cone-beam computed tomography image. Note the opacity of the left sinus.

**Figure 3:**
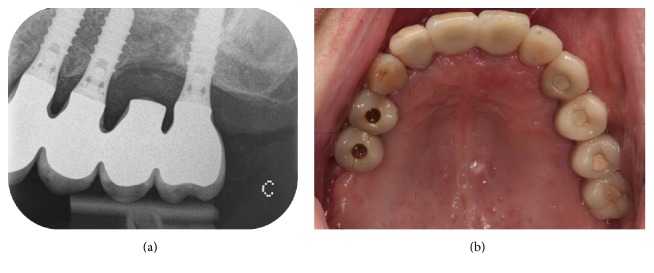
The implant 25 was removed and the bridge was rescrewed. The patient underwent antibiotic treatment. (a) Periapical radiograph and (b) clinical view.

Ten years later, on an annual recall in 2013, the patient underwent a routine implant and periodontal follow-up examination. This appointment was not asked for by the patient who did not complain about specific intraoral symptoms, pains, or adverse events. She described only a slight painless discomfort in the left infraorbital region that had lasted for a few months. She showed no extraoral symptoms of sinusitis. The probing pocket depth was measured at six locations around the implants. The values were between 4 and 7 mm for the implants 23, 24, and 26. They were between 6 and 10 mm for the implant 25. The total implant length was 11.8 mm, including the implant collar. Peri-implantitis was diagnosed. Radiography showed crestal bone loss around implant 25 (Figure 2(a)). A flap was elevated to explore the site. It revealed that the bone loss reached the implant apex (Figure 2(b)). Cone-beam computed tomography (CBCT; Model CS 9300, Carestream Health, Inc., Rochester, New-York, USA) showed an opaque left sinus (Figure 2(c)). The FPD was unscrewed, revealing the mobility of implant 25. The failed implant got out spontaneously when the bridge was removed (Figures 3(a)-3(b)). An OAC was identified clinically at site 25. The FPD was rescrewed and antibiotics (Dalacin® C, Pfizer, Zürich, Switzerland; 300 mg, 3 times per day for 5 days) were administered to the patient

**Figure 4:**
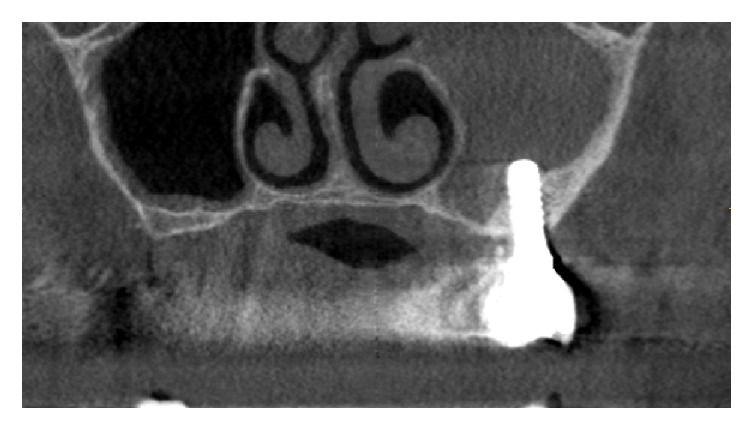
Six months after the removal of the implant 25. The cone-beam computed tomography image revealed that the opacity of the left sinus was still present.

**Figure 5:**
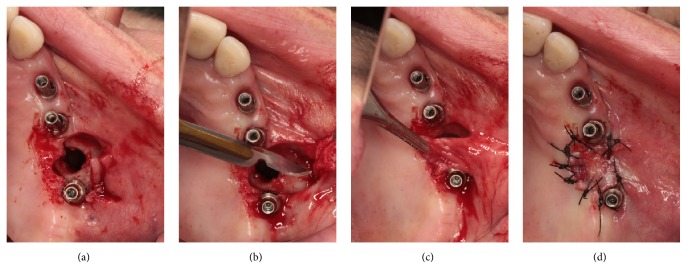
The oroantral fistula was closed with a buccal advancement flap. (a)–(d) Clinical views.

**Figure 6:**
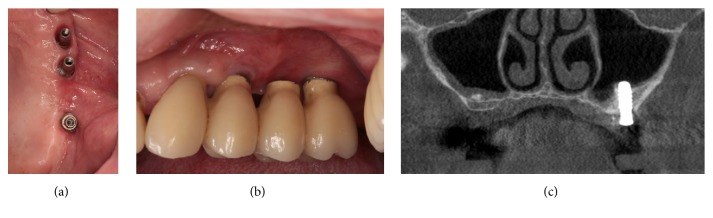
Two months after the closure of OAF. (a)-(b) Clinical views. The oroantral fistula remained successfully closed. (c) Cone-beam computed tomography image. The left sinus was totally healed.

**Figure 7:**
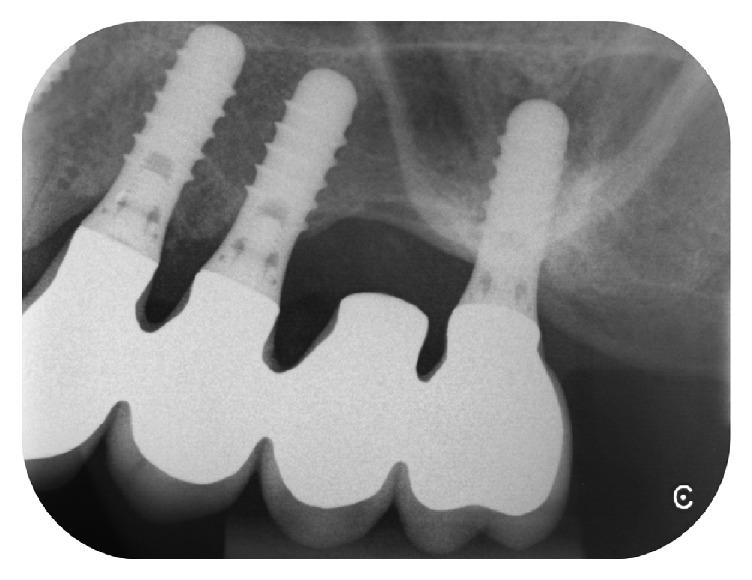
Two years after the closure of OAF; the radiographic control showed a stable crestal bone level.

Six months later, persistence of the sinus opacity was observed on CBCT (Figure 4) and an OAF was then formed. The patient still had not complained about any sinus symptoms. The FPD was unscrewed, the sinus was irrigated and rinsed through the fistula with NaCl and H_2_O_2_ solutions, and the FPD was rescrewed. The procedure was repeated once a week for six weeks, until the sinus showed no pus and inflammatory exudates during rinsing. The OAF was closed with a buccal advancement flap under antibiotic therapy initiated the day prior to surgery (Dalacin; Figures 5(a)–5(d)). A nasal spray (Otrivin®, GSK Consumer Healthcare Schweiz AG, Rotkreuz, Switzerland) was administered to the patient (3 times per day for 6 days). The sutures were removed after two weeks and the FPD was rescrewed. After an uneventful healing period of two months, the FPD was unscrewed. Clinical examination showed that the OAF remained successfully closed (Figures 6(a)-6(b)), and CBCT images confirmed total healing of the sinus (Figure 6(c)). Two years later, the bridge was clinically stable and the probing pocket depth was <3 mm for the implants 23, 24, and 26. Radiographic control showed that the crestal bone level was stable in this area (Figure 7).

## 3. Discussion

The incidence rate of sinusitis after sinus elevation procedure was estimated to about 12% when a lateral approach is performed [5]. Most of sinusitis (84.8%) occurred within 3 weeks after sinus elevation procedure [5]. The sinus graft infections as a result of peri-implantitis are a major acute complication and necessitate urgent treatment [12]. Histological examination showed that bacteria were present inside the sinus, along mainly the biomaterial grafted particles and also the newly formed bone [12].

The case reported in this article did not have to be treated as an emergency. The sinusitis did not present as acute but was chronic and asymptomatic. No clinical signs of sinusitis or peri-implantitis were reported by the patient. The adverse event was discovered during a routine control, 10 years after implant placement. This article reveals the difficulty in diagnosing one implant failure and OAC under a stable bridge supported by four implants without intraoral and extraoral clinical symptoms. The bone loss around only one implant was detected during examination by measurement of the pocket depth around the implant and by further radiography. No other clinical signs, such as bleeding and/or suppuration on probing, were visible. Given that the implant was one the four supports for a splinted multiunit FPD, mobility of the implant could not be observed. This led first to a diagnosis of peri-implantitis, although it was atypical [13]. In addition, because of the narrow peri-implant bone lesion and the presence of implant spires, the true extent of clinical probing depth was underestimated; the OAC could not be clinically diagnosed at this time.

The presence of chronic sinusitis was diagnosed later by the use of CBCT. The CBCT is more specific and sensitive for analysis of the degree of sinus abnormalities than standard periapical and panoramic radiographs [14]. When the sinus is affected, opacity of the sinus and thickening of the Schneiderian membrane are usually observable on tomography. In the present case, the CBCT examination has supported the presence of sinus pathology. However, although it revealed unilateral sinusitis, it did not detect the OAC. The difficulty in observing the discontinuity of the bony floor of the maxillary sinus may have been due to a high level of image noise [15].

Unscrewing of the FPD was needed to identify the implant mobility and OAC. The progressive crestal bone loss and OAC may have predisposed implant failure and further induced OAF. The management of the OAF was standard. It included removal of the implant, antibiotic therapy, and abundant rinsing. The technique used for the surgical closure of the OAF was identical to that used for the treatment of OAF occurring after the extraction of maxillary molars, that is, soft tissue closure using a buccal or palatal flap. This procedure is the most frequently used; it is quick, safe, straightforward, and well tolerated by patients [16]. At its removal, the implant did not present residual inflammatory tissues on its surface. Surgical curettage of the maxillary sinus was not considered. The radiological signs of sinusitis did not disappear after implant removal but after the surgical closure of the OAF. At the end of the treatment, the patient retained the same FPD in a healthy oral condition.

In the present article, the long-term infection of the grafted material was debatable but not relevant. The complication involved only one implant and, in addition, it was expected that, after 10 years, the deproteinized bovine bone material used for the grafting was well integrated in lamellar bone, with intact and nonresorbed particles [17]. Individual predisposition of the patient to the periodontitis, although treated and stabilized before implant surgery, might have influenced the rate of bone loss. The peri-implantitis and OAC were consequent on the progressive crestal bone loss around the osseointegrated implant.

Maxillary sinusitis of dental origin is unilateral [18]. It typically develops in association with reduced drainage of the maxillary sinus. Perforation of the maxillary sinus membrane can lead to sinus complications, which most often occur within the first few weeks after surgery [9]. Intrusion of the implant into the sinus floor can give rise to sinusitis or rhinosinusitis, but this occurs generally in patients with a predisposition for sinusitis [19]. Sinus complications can be also related to the presence of a foreign body in the sinus, such as a mobile implant or bone grafting material that has migrated during surgery [20]. Few weeks after implant surgery, sinus complications are generally associated with non-osseointegrated implants, which maintain an OAC. This leads to the early failure of the implants. Only a single case of late failure of implants under a bridge, along with sinusitis, was reported in the literature [21]. Five years after implant placement, the diagnosis was immediate and unambiguous because the patient showed significant clinical symptoms of sinusitis—gingival swelling and abscess formation—as well as radiological signs. On removal of bridges, mobility of all the implants was detected and OAF was observed [21]. To the authors' knowledge, long-term failure of one implant under a stable bridge has not been reported elsewhere.

## 4. Conclusions

This report has shown that late loss of implant osseointegration in the posterior maxilla can be the cause of an OAC. It reflects the difficulty in diagnosing an OAC following late failure of a single implant under a multiunit FPD and the importance of sinus check-up during a routine implant examination. Unilateral radiopacity of the maxillary sinus in the presence of posterior dental implants may indicate implant failure and underlying OAC.
